# Feasibility and acceptability of ice plant intensive cream in preventing hand-foot syndrome in breast cancer patients undergoing Doxorubicin and/or Docetaxel therapy: a randomized controlled pilot study

**DOI:** 10.3389/fonc.2026.1758165

**Published:** 2026-08-31

**Authors:** Marcela Winkler, Renée Arnhold, Dennis Anheyer, Christel Idler, Felicitas Löffler, Georg Sauer, Claudia Löffler, Holger Cramer

**Affiliations:** 1 Department of Integrative Medicine, Robert Bosch Hospital, Stuttgart, Germany; 2 Robert Bosch Center for Integrative Medicine and Health (RBIM), Robert Bosch Medical Research Society (RBMF), Bosch Health Campus (BHC), Stuttgart, Germany; 3 Medical Faculty, University of Tübingen, Tübingen, Germany; 4 Institute of General Practice and Interprofessional Care, University Hospital Tübingen, Tübingen, Germany; 5 Department of Gynecology, Robert Bosch Hospital, Stuttgart, Germany; 6 Department of Internal Medicine II, University Hospital of Wuerzburg, Wuerzburg, Germany

**Keywords:** breast cancer, chemotherapy-induced toxicity, Docetaxel, Doxorubicin, feasibility, hand-foot syndrome, ice plant, oncology supportive care

## Abstract

**Introduction:**

Hand-Foot Syndrome (HFS) is a common side effect in patients undergoing chemotherapy, particularly with Doxorubicin and Docetaxel and can substantially impair quality of life. This study aimed to assess the feasibility and acceptability of using Ice Plant Intensive Cream (*Mesembryanthemum crystallinum*) to prevent HFS in breast cancer patients, within a predefined recruitment target of 36 participants.

**Methods:**

In this randomized controlled pilot study, patients with breast cancer scheduled to receive treatment with doxorubicin or docetaxel were enrolled. Participants were randomly assigned either to an intervention group, which followed standard HFS prevention plus Ice Plant Intensive Cream to the hands and feet three times daily during chemotherapy, or a control group that received standard HFS prevention alone. Feasibility endpoints included recruitment rate, inclusion rate, retention, adherence to the intervention, and completeness of data collection. Secondary exploratory endpoints included HFS severity, skin-related symptoms, and quality of life.

**Results:**

Between January and November 2023, 330 patients were screened during tumor board meetings, of whom 58 met the eligibility criteria. Ultimately, 13 patients (22.4% of eligible) consented and were enrolled, indicating that neither the predefined recruitment target nor the randomization feasibility criterion were met. Most non-participation was attributable to patient overload during the diagnostic phase or initiation of chemotherapy before contact. Once enrolled, feasibility metrics related to retention, adherence, and data completeness were achieved, with a dropout rate of 15%, protocol adherence of 100% among participants who completed the study, and more than 90% completeness of scheduled assessments.

**Conclusion:**

The overall study was not feasible as designed, primarily due to recruitment limitations. However, once patients were enrolled, intervention procedures, data collection, and protocol adherence were fully feasible. Optimization of recruitment timing and earlier patient engagement during the diagnostic phase are recommended for future confirmatory trials.

**Clinical Trial Registration:**

https://clinicaltrials.gov/study/NCT05755646, identifier NCT05755646.

## Introduction

Hand-Foot Syndrome (HFS) is a frequent and disruptive side effect experienced by patients undergoing chemotherapeutic treatments, particularly those receiving agents such as Doxorubicin and/or Docetaxel (1, 2). Chemotherapy remains a cornerstone in breast cancer treatment, especially for patients with aggressive subtypes such as triple-negative and HER2-positive disease. According to current guideline-based treatment pathways, anthracycline- and/or taxane-containing regimens remain among the most commonly recommended systemic options for patients with early or locally advanced breast cancer who are at higher risk of recurrence (3–5).

HFS manifests with erythema, swelling, pain and in severe cases with blistering or ulcerations on the palms and soles, leading to substantial impairment of quality of life (QoL) and treatment adherence (1, 2). These symptoms often lead to functional limitations in everyday life, for example difficulties in holding or grasping objects with the hands (6). In severe cases, HFS may necessitate dose reductions or discontinuation, potentially compromising therapeutic efficacy (1).

Breast cancer patients, especially those undergoing combination chemotherapy regimens, are particularly vulnerable to HFS, with incidence rates ranging from approximately 22% to 63% for doxorubicin-, docetaxel-, or combination-based therapies, and up to 89% in regimens containing 5-fluorouracil, depending on drug type and dose intensity (2, 7). In adjuvant settings, capecitabine has shown some of the highest rates, with 73% of patients affected in the CREATE-X trial, while lower-dose metronomic schedules, such as in SYSUCC-001, resulted in reduced but still considerable rates around 45% (7, 8). These observations illustrate the strong influence of drug type and dosing schedule on the development of HFS.

Notwithstanding its high clinical relevance, there is no universally accepted standard for the prevention or management of HFS. Current supportive-care guidelines primarily recommend alleviating measures such as 5-10% urea-based topical emollients and physical interventions to reduce mechanical stress and heat exposure, though the supporting evidence remains limited (4, 9). Although some evidence suggests a positive association between the occurrence of HFS and improved progression-free and overall survival (2), this toxicity remains a substantial burden that negatively affects patient well-being and adherence to therapy. Therefore, designing supportive strategies to prevent or alleviate HFS symptoms remains clinically important.

Several randomized trials have demonstrated that topical preventive strategies can significantly reduce the incidence and severity of chemotherapy-induced skin toxicities, establishing supportive oncodermatology as a clinically relevant field (10–12). However, preventive data specifically on anthracycline- and taxane-induced HFS remain lacking.

*Mesembryanthemum crystallinum*, commonly known as ice plant, is a succulent that thrives under high-stress environments such as arid or saline soils. It has long been used in dermatological care for its hydrating and soothing properties. Early laboratory and clinical observations suggest that extracts of ice plant improve skin hydration and barrier function, which may reduce inflammatory responses and alleviate symptoms of various dermatological conditions, including HFS (13). These properties may counteract the skin stress and inflammatory mechanisms associated with chemotherapy-induced HFS. Although existing evidence is preliminary, it provides a rationale for exploring the feasibility of preventive use of ice plant in oncology settings. This pilot study therefore aimed to assess the feasibility and acceptability of using Ice Plant Intensive Cream as a supportive intervention to prevent chemotherapy-induced HFS in breast cancer patients. The results aim to provide methodological insights into recruitment, adherence, and study logistics, forming a basis for improving the design and implementation of future feasibility-oriented research.

## Methods

### Study design and setting

This study was a prospective, randomized controlled pilot trial evaluating the feasibility of the topical application of *Mesembryanthemum crystallinum* (Ice plant) Intensive Cream in the prevention of Hand-Foot Syndrome (HFS) in breast cancer patients undergoing chemotherapy with Doxorubicin and/or Docetaxel. The study was registered at ClinicalTrials.gov prior to patient recruitment (NCT05755646). Ethical approval was granted by the Ethics Committee of the State Medical Chamber of Baden-Württemberg (Landesärztekammer Baden-Württemberg), project number F-2022-118, on 10th October 2022. The study is reported in accordance with the Consolidated Standards of Reporting Trials (CONSORT) statement and its extension to randomized pilot and feasibility trials (14).

The primary outcome was the overall feasibility of implementing the study procedures, assessed across key feasibility domains including patient recruitment, inclusion rate, adherence, and completion rates.

Feasibility was defined according to the following pre-specified criteria (**Table 1**):

**Table 1:** Feasibility criteria and success thresholds.

Domain	Definition	Success criterion
Recruitment	Eligible patients enrolled within 9 months.	≥ 36 patients recruited.
Randomization	Eligible patients consenting to randomization.	≥ 50% randomized.
Retention	Participants completing all visits.	≤ 20% dropout.
Adherence	Cream use as prescribed.	≥ 80% protocol adherence.
Data completeness	Available data at all time points.	< 25% missing DLQI, < 30% diary.
Assessment feasibility	Duration and practicality of data collection.	Average < 1 h per visit.
Acceptability	Satisfaction with procedures/intervention.	≥ 70% rated “good” or better.

- Recruitment feasibility: achieved if the required sample of n=36 patients could be recruited within 9 months.
- Randomization feasibility: achieved if at least 50% of the potentially eligible patients agree to participate and were successfully randomized.
- Attrition feasibility: achieved if dropout rate per group did not exceed 20%.
- Intervention feasibility: achieved if at least 80% of participants completing the study received treatment according to protocol.
- Assessment feasibility: achieved if (i) data collection per participant and time point required less than one hour on average and (ii) for patients completing the study, data loss was < 25% for the dermatology-specific quality of life (DLQI) and < 30% for the study diary.

Secondary exploratory outcomes included the incidence and severity of HFS, changes in DLQI, and patient-reported outcomes related to pain, skin symptoms, and functional limitations.

### Patients and recruitment

Patients with a confirmed diagnosis of breast cancer scheduled to undergo chemotherapy with Doxorubicin and/or Docetaxel, were recruited by study physicians and staff at the Robert Bosch Hospital in Stuttgart, Germany. Eligible patients were required to be at least 18 years old and to have a Karnofsky Performance Status (KPS) score above 80. Exclusion criteria included pre-existing dermatologic conditions affecting the hands or feet, peripheral neuropathy, or known hypersensitivity to any component of the Ice Plant Intensive Cream. Patients who had already initiated chemotherapy prior to study enrollment or had previously used the investigational cream were also excluded.

All potential participants received a comprehensive verbal and written explanation of the study procedures from trained research staff. Written informed consent was obtained from all participants before enrollment, in accordance with the Declaration of Helsinki and applicable data protection regulations. Participants were informed that participation was voluntary and that consent could be withdrawn at any time during or after the study without any impact on their ongoing medical care.

### Randomization and blinding

After providing verbal and written informed consent, participants were randomized in a 1:1 ratio to either the intervention group (Ice Plant Intensive Cream plus standard care) or the control group (standard care only) via block randomization with randomly varying block length. The randomization sequence was created by an external scientist who had no participant contact during the entire study using R software (version 4.3.1). The randomization list was kept password-protected and only the scientist creating the list had access to it. Randomization was performed by telephone through the Center for Clinical Studies at the Robert Bosch Hospital in Stuttgart, Germany after verification of eligibility criteria and receipt of written informed consent. The group allocation was communicated to the study site by phone and documented in the trial records. Due to the visible nature of the intervention, blinding of participants and investigators was not possible.

### Intervention

All participants received standard preventive care for hand–foot syndrome (HFS) according to supportive-care recommendations, including general skin care advice and regular use of a 5–10% urea-based cream on hands and feet. Participants in both groups were advised to avoid additional topical products on the hands and feet and to minimize mechanical stress during the study period.

Participants randomized to the intervention group additionally applied Ice Plant Intensive Cream (Mesembryanthemum crystallinum, Dr. Hauschka Med, EU KVO Nummer 1223/2009, WALA Heilmittel GmbH, Bad Boll, Germany) to the palms and soles three times daily. Applications took place in the morning, at noon, and in the evening. Patients spread a thin layer and gently massaged until absorbed leaving it on the skin for at least 30 minutes without wiping or washing it off. The intervention started on the first day of chemotherapy and continued throughout all cycles until seven days after the final cycle.

Study staff provided the cream at each study visit and gave verbal and written instructions for correct use. Adherence was captured in a daily patient diary that documented each application, HFS symptoms graded with the WHO scale, pain and sensory discomfort using a 0–10 numeric rating scale (NRS), and any concomitant medication. Cooling procedures performed during chemotherapy were permitted as per routine care and recorded in the diary.

Safety monitoring included continuous assessment of local skin reactions and adverse events during chemotherapy appointments and follow-up visits. The team recorded all adverse events (AEs) and serious adverse events (SAEs) and reported them according to Good Clinical Practice (GCP) standards and the approved protocol.

### Outcomes and measures

The primary outcome of this pilot trial was the overall feasibility of implementing the study procedures in preparation for a larger confirmatory trial. Feasibility was evaluated across the domains of recruitment, randomization, adherence, data completeness, and dropout rates.

Secondary exploratory outcomes focused on the incidence and severity of HFS, dermatology-related quality of life, pain, and patient-reported skin symptoms.

HFS severity was graded according to the World Health Organization (WHO) (9) classification, distinguishing four levels:

Grade 1 = mild erythema or dysesthesia without functional limitation;

Grade 2 = erythematous swelling with pain on pressure;

Grade 3 = painful swelling and fissures limiting daily activities;

Grade 4 = severe pain with blistering or ulceration preventing daily activities.

Grading was self-reported by patients in their study diaries and verified by study staff at each visit.

Pain and sensory discomfort were assessed using a 0–10 Numeric Rating Scale (NRS) (15), documented daily in the patient diary to monitor symptom progression during chemotherapy cycles.

Dermatology Life Quality Index (DLQI) (16): This validated instrument measured dermatology-specific quality of life, covering symptoms, functional limitations, and emotional well-being. The DLQI was administered at baseline and at each predefined assessment point.

Concomitant medication and supportive care: Patients recorded all concomitant medications (excluding chemotherapy), additional topical products, and any local cooling procedures performed during chemotherapy.

Adherence and acceptability: Participants documented the frequency of Ice Plant Intensive cream application, any local skin reactions, and side effects daily in their diaries. Adherence to the intervention protocol was defined as cream application at least twice daily on five or more days per week. In the intervention group only, patient-reported acceptability and tolerability of Ice Plant Intensive Cream were assessed using the M-Questionnaire (13 items). The instrument captures perceived skin effects, usability, and overall tolerability. Responses were recorded on 5-point ordinal scales, and analyses were descriptive only. (see **Supplementary Material 1** for the questionnaire).

### Data collection and assessment schedule

**Figure 1:**
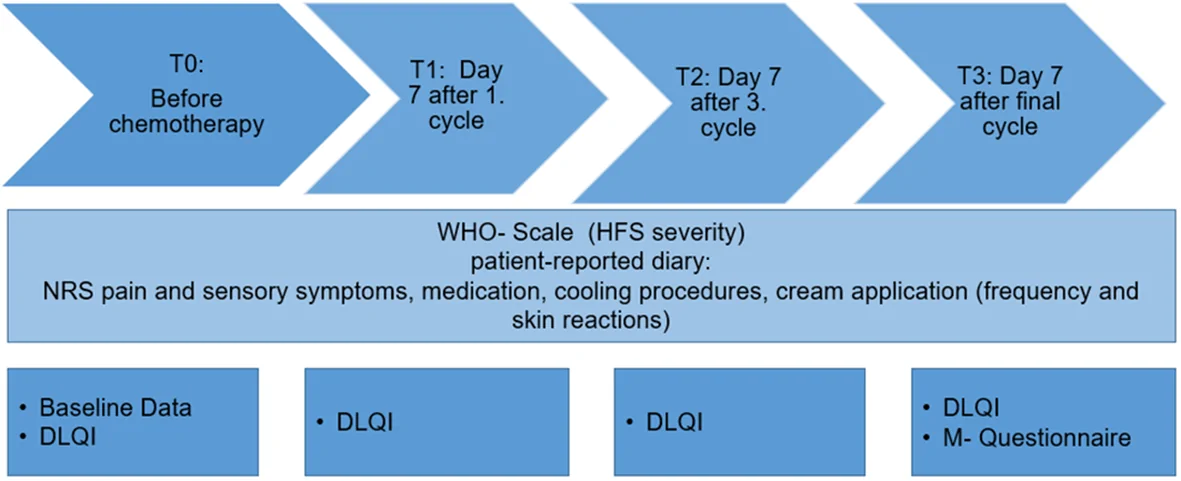
Assessment schedule and outcome measures across all study time points (T0–T3).

Data collection followed a predefined schedule across four time points: T0 = baseline (before the start of chemotherapy), T1 = seven days after the first cycle, T2 = seven days after the third cycle, and T3 = seven days after the final chemotherapy cycle, as illustrated in **Figure 1**.

At each time point, patients completed the Dermatology Life Quality Index (DLQI), self-rated Hand–Foot Syndrome severity using the WHO-HFS grading scale, and recorded pain intensity, sensory disturbances and general impairment of daily life using the 0–10 Numeric Rating Scale (NRS).

Participants documented daily use of Ice Plant Intensive Cream, local skin reactions, and any adverse events in a paper-based patient diary provided by the study team. The diaries were reviewed and collected by study staff during hospital visits, and relevant entries were transferred into the electronic study database after verification for completeness and accuracy.

In addition, study staff maintained regular contact with participants by telephone and e-mail to provide reminders for study visits, clarify questions about cream application or diary entries, and ensure participant safety between chemotherapy cycles and scheduled assessments.

### Sample size calculation and statistical analysis

The primary outcome of this pilot study was the feasibility of conducting a larger confirmatory RCT.

The planned confirmatory study is intended to be powered to detect a 50% reduction in incidence with a power of 80%. Based on an expected incidence of 50% in the control group and an expected incidence of 25% in the intervention group, an exact Fisher test with a power of 80% requires a sample size of n=128 patients. Taking into account a dropout rate of up to 10%, the confirmatory study will require a total of n=144 patients. The pilot study was planned with a fixed number of patients equal to 25% of the patients required for the confirmatory study (total n=36 patients).

The data was initially entered and collected in Microsoft Excel (version 2021; Microsoft Corp., Redmond, WA, USA). Descriptive statistical analyses were performed using JASP (version 0.18.3; University of Amsterdam, Amsterdam, Netherlands). Figures and tables were created using JASP, Microsoft Word.

Descriptive statistics, including means, standard deviations, medians, and interquartile ranges, were used to summarize these feasibility measures and the secondary outcomes. This pilot trial was exploratory in nature and was not powered or designed for confirmatory hypothesis testing.

## Results

### Patient characteristics

All study participants were female and diagnosed with breast cancer. The median age in the intervention group was 42 years (range: 33-53), while in the control group it was 50 years (range: 45–61). (see **Table 2**).

**Table 2:** Patient characteristics.

Characteristic	Control group (CG)	Intervention group (IG)	Total
Number of Participants	n= 6 (54.5%)	n=5 (45.5%)	n=11
Age, median (range)	50 (45–61)	42 (33–53)	46 (33–61)
Comorbidities, n (%)			
Arterial hypertension	3 (50)	1 (20)	4 (36.4)
Anxiety disorder	1 (16.7)	–	1 (9.1)
Pulmonary embolism	1 (16.7)	–	1 (9.1)
Chemotherapy-Regimen, n (%)			
*12× Paclitaxel + 4× AC*	1 (16.7)	2 (40)	3 (27.3)
*6×Docetaxel/Cyclophosphamid*	2 (33.3)	1 (20)	3 (27.3)
*4×Paclitaxel/Carboplatin + 4× AC*	2 (33.3)	0	2 (18.2)
*6× Docetaxel/* *Carboplatin*	1 (16.7)	2 (40)	3 (27.3)
average duration	129.5 days	133.8 days	

Most patients received anthracycline- and/or taxane-based combination protocols, including doxorubicin, cyclophosphamide, paclitaxel, and docetaxel, either alone or in combination with carboplatin. Detailed chemotherapy regimens, dosing schedules, and treatment duration for each group are presented below in **Table 2**.

### Participant flow

A total of 330 potential candidates were identified during tumor board discussions between January and November 2023 at the Robert Bosch Hospital, Stuttgart. Of these, 58 patients (17.6%) fulfilled the eligibility criteria and were contacted by the study team for detailed screening. Thirteen patients (22.4% of eligible) provided written informed consent and were randomized to the intervention (n=6) or control group (n=7).

Of the 58 eligible patients, the most common reasons for non-participation included chemotherapy-related issues such as refusal of chemotherapy, change to the originally indicated chemotherapy regimen, or chemotherapy already initiated prior to possible study inclusion (n=17). The second most common reason for non-participation was a lack of interest in the study, mostly due to emotional overload immediately after diagnosis (n=16). Less common reasons were unsuccessful attempts to make contact, oncologists advising against participation in the study, and a lack of German language proficiency (n=5). A further seven patients were excluded after giving preliminary consent during the informational meeting, because by the time they were called in to sign the final consent and undergo formal enrollment, chemotherapy had already started.

One participant in each group discontinued the study prematurely, leading to dropout rates of 14.3% in the CG and 16.7% in the IG. One participant who was retrospectively excluded due to incorrect inclusion nevertheless completed all planned examinations. In the second case, the patient was enrolled but declined participation, as her oncologist did not consider her to be at risk of HFS.

Overall, 12 of 13 participants (92.3%) completed all planned study visits and assessments.

**Figure 2:**
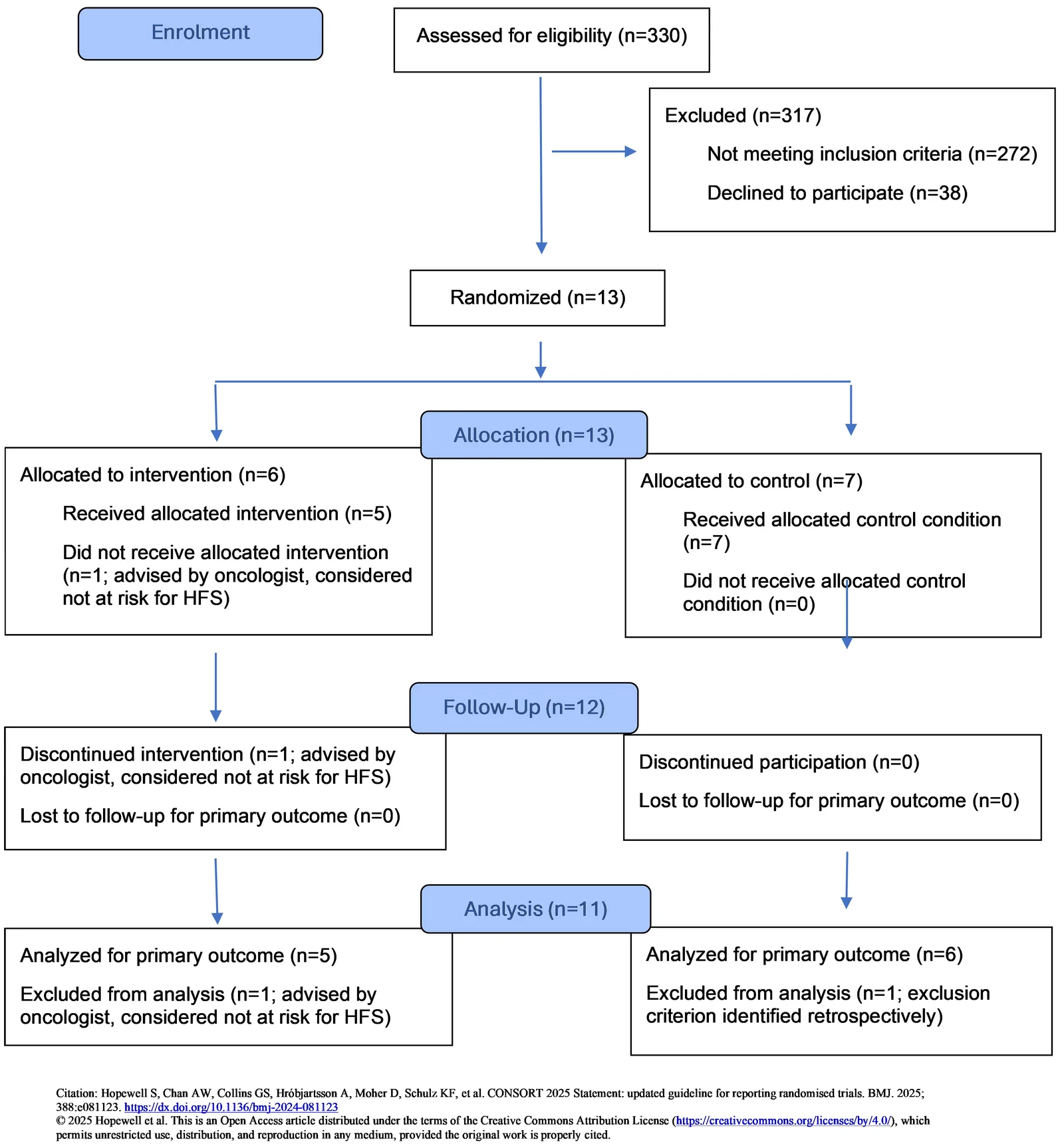
Consort 2025 flow diagram. Flow diagram of the progress through the phases of a randomised trial of two groups (that is, enrolment, intervention allocation, follow-up, and data analysis).

A detailed overview of recruitment, randomization, completion, and assessment to protocol is shown in the CONSORT Flow Diagram (**Figure 2**).

### Feasibility outcomes

**Figure 3:**
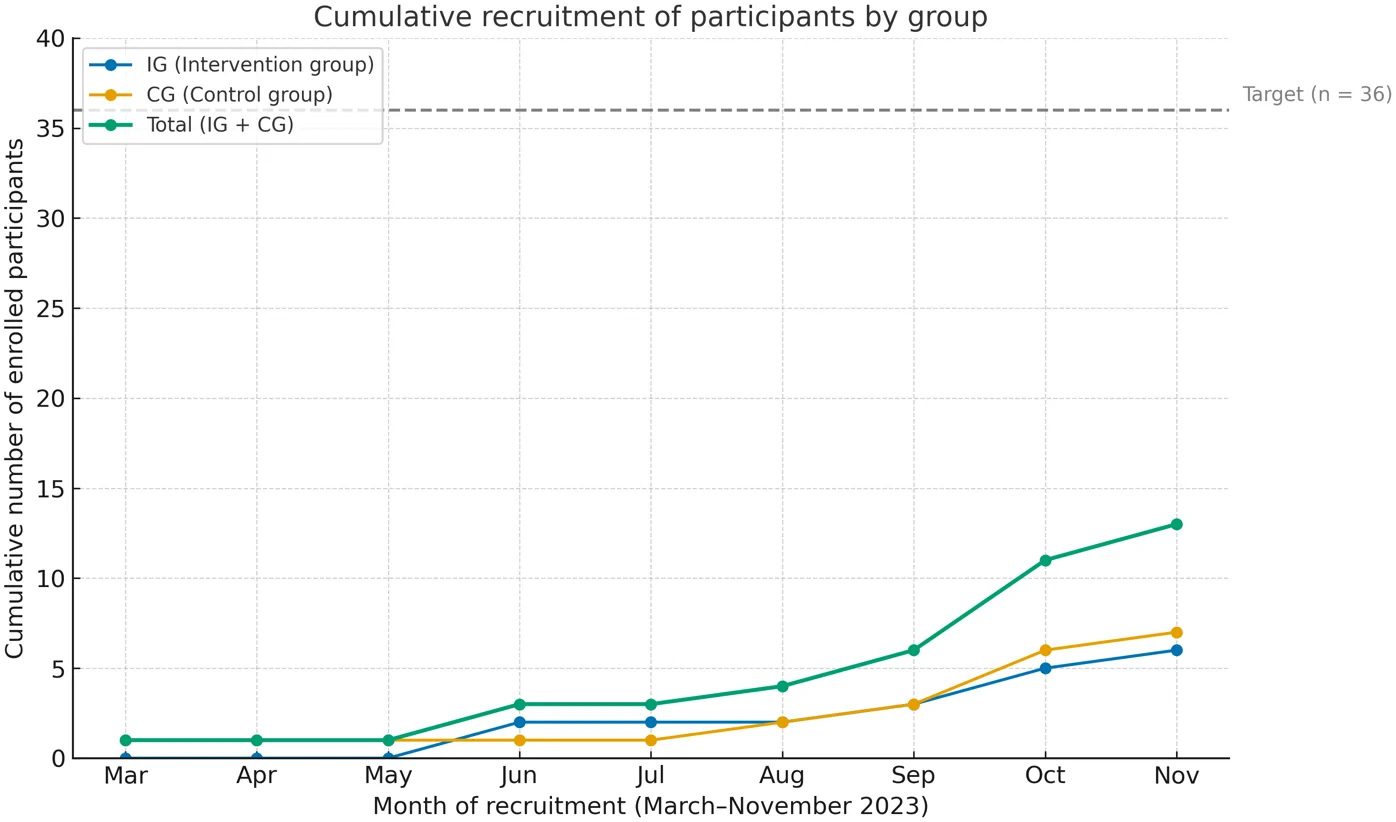
Monthly cumulative recruitment of participants in the intervention and control group.

Over the recruitment period, 330 patients were discussed in the tumor board at Robert Bosch Hospital, Stuttgart. Among them, 67 (20.3%) were scheduled to receive chemotherapy with doxorubicin and/or docetaxel and were considered potentially eligible. After detailed screening, 58 patients (17.6% of all screened) fulfilled the inclusion criteria and were contacted by telephone for study information. Of these, 13 patients (22.4% of eligible, 3.9% of all screened) provided written informed consent and were randomized to the intervention group (n=6) or the control group (n=7). **Figure 3** illustrates the cumulative recruitment over the study period. Despite continuous screening, the predefined recruitment target of 36 patients was not achieved. The main barriers to participation were emotional and organizational constraints.

As a result, a total of 12 out of 13 participants (92.3%) completed all study visits and examinations, thereby fulfilling the predefined feasibility criterion for compliance (≥80% protocol compliance). Adherence to the intervention protocol—defined as cream application at least twice daily on five or more days per week—was achieved by all participants who completed the study. Data completeness for the DLQI and symptom diary exceeded 80% across all time points, confirming the feasibility of data collection. With regard to the feasibility of the assessment, the goal was also achieved, as the duration per assessment point was consistently less than 30 minutes (cf. **Tables 3**, **4**).

**Table 3:** Feasibility of data collection (excluding drop-outs).

Criterion	Target	Result	Evaluation
Average time per assessment	<60 minutes	100% <30 minutes	Feasible
Missing data	<20%	<10%	Feasible

**Table 4:** Completeness of assessments across all time points (including drop-outs).

Assessment instrument	T0	T1	T2	T3	Complete (n=13)
Baseline data	12	–	–	–	12
DLQI	12	12	12	12	12
WHO Scale	12	12	12	12	12
M Questionnaire (Intervention group only; n=6)	–	–	–	5	5
Diary	12	12	12	12	12

Randomization was technically feasible and successfully implemented by the Center for Clinical Studies via telephone; however, only 22.4% of eligible patients agreed to participate, and thus the predefined randomization feasibility criterion (≥ 50%) was not achieved.

**Table 5** summarizes all feasibility outcomes in relation to the predefined success criteria.

**Table 5:** Feasibility outcomes relative to predefined criteria.

Domain	Definition	Success criterion	Observed result	Criterion met
Recruitment	Eligible patients enrolled within 9 months.	≥ 36 patients recruited.	13 patients recruited (36.1% of target).	✗ Not met
Randomization	Eligible patients consenting to randomization.	≥ 50% randomized.	22.4% (13 of 58 eligible).	✗ Not met
Retention	Participants completing all visits.	≤ 20% dropout per group.	CG: 14.3% (1 of 7) IG: 16.6% (1 of 6).	✓ Met
Adherence	Cream use as prescribed.	≥ 80% protocol adherence.	100% (5 of 5 completed ≥ 2×/day ≥ 5 days/week).	✓ Met
Data completeness	Available data at all time points.	< 25% missing DLQI, < 30% missing diary.	> 80% data complete.	✓ Met
Assessment feasibility	Duration and practicality of data collection.	Average < 1 h per visit.	Achieved (< 30 min per assessment).	✓ Met
Acceptability	Satisfaction with procedures/intervention.	≥ 70% rated “good” or better.	83% reported high satisfaction.	✓ Met

Overall, the study procedures proved to be safe, well tolerated, and acceptable, whereas the recruitment rate remained the major limiting factor for conducting a larger confirmatory trial under current clinical conditions.

### Exploratory clinical outcomes

Descriptive analyses were conducted for all exploratory outcomes. Where appropriate, means, standard deviations, medians and interquartile ranges were calculated. No inferential statistical tests were applied, due to the exploratory and feasibility-focused nature of this pilot study.

Based on the mean grade per participant, no grade 3 or 4 Hand-Foot Syndrome (HFS) was observed during the study period; one participant in the intervention group recorded grade 3 and grade 4 on individual days. Mild erythema or dysesthesia corresponding to WHO grade 1, occurred in three participants (27.3%), and moderate erythema or swelling (grade 2) was the highest grade recorded in one case (9.1%). Apart from this participant, no ulcerative lesions or severe functional impairments were reported. The distribution of HFS severity grades was similar between the intervention and control groups. Detailed descriptive statistics for HFS severity and distribution are summarized in (**Table 6**).

**Table 6:** Exploratory clinical outcomes: hand-foot syndrome severity and patient-reported symptoms (analysis population, n = 11).

	Intervention group (n=5)			Control group (n=6)		
Outcome	Mean (SD)	Median (IQR)	Min–Max	Mean (SD)	Median (IQR)	Min–Max
Per-participant mean diary scores over the treatment period						
HFS severity, WHO grade (0–4)	0.5 (0.9)	0.05 (0.3)	0.0–2.2	0.2 (0.3)	0.0 (0.2)	0.0–0.8
NRS pain, hands (0–10)	1.8 (1.8)	1.0 (0.06)	1.0–5.1	1.5 (1.2)	1.0 (0.09)	1.0–3.9
NRS pain, feet (0–10)	1.6 (1.2)	1.0 (0.2)	1.0–3.6	1.4 (1.1)	1.0 (0.0)	1.0–3.6
NRS sensory disturbance, hands (0–10)	1.5 (0.9)	1.2 (0.4)	1.0–3.0	1.6 (1.3)	1.0 (0.4)	1.0–4.2
NRS sensory disturbance, feet (0–10)	1.2 (0.1)	1.2 (0.1)	1.1–1.4	1.5 (1.1)	1.0 (0.2)	1.0–3.8
NRS impairment of daily life, hands (0–10)	1.9 (1.7)	1.0 (0.4)	1.0–4.9	1.5 (1.1)	1.0 (0.2)	1.0–3.7
NRS impairment of daily life, feet (0–10)	1.5 (1.0)	1.0 (0.2)	1.0–3.2	1.4 (1.0)	1.0 (0.0)	1.0–3.5
Highest WHO grade recorded per participant, n (%)						
Grade 0	2 (40.0)			4 (66.7)		
Grade 1	2 (40.0)			1 (16.7)		
Grade 2	0			1 (16.7)		
Grade 3	0			0		
Grade 4	1 (20.0)			0		

HFS, hand-foot syndrome; IQR, interquartile range; NRS, numeric rating scale; SD, standard deviation; WHO, World Health Organization. Diary entries were recorded daily from the first day of chemotherapy until seven days after the final cycle. For each participant, the mean of all valid daily entries was calculated; missing entries were excluded. Group statistics are based on these per-participant means. The grade distribution reports the highest WHO grade documented on any single day. Based on the mean grade per participant, no participant reached grade 3 or 4; one participant in the intervention group recorded grade 3 and grade 4 on individual days. No inferential statistical tests were performed.

Regarding dermatology-specific quality of life, the mean Dermatology Life Quality Index (DLQI) score at baseline was low, indicating minimal dermatological burden at study entry. Across subsequent assessment points, DLQI values showed only small fluctuations, with no clinically relevant deterioration. A descriptive overview of mean DLQI scores, standard deviations, and interquartile ranges for all time points (T0–T3) is presented in (**Table 7**).

**Table 7:** Dermatology Life Quality Index (DLQI) total score at the four assessment time points (analysis population, n = 11).

	Intervention group (n=5)			Control group (n=6)		
Time point	Mean (SD)	Median (IQR)	Min–Max	Mean (SD)	Median (IQR)	Min–Max
T0 (baseline)	0.0 (0.0)	0.0 (0.0)	0–0	0.0 (0.0)	0.0 (0.0)	0–0
T1	1.0 (2.2)	0.0 (0.0)	0–5	1.0 (2.4)	0.0 (0.0)	0–6
T2	1.0 (1.0)	1.0 (2.0)	0–2	1.2 (1.9)	0.5 (1.0)	0–5
T3	1.2 (2.7)	0.0 (0.0)	0–6	0.7 (1.6)	0.0 (0.0)	0–4

DLQI, Dermatology Life Quality Index (total score range 0–30; higher scores indicate greater impairment); IQR, interquartile range; SD, standard deviation. T0 = baseline before the start of chemotherapy; T1 = seven days after the first cycle; T2 = seven days after the third cycle; T3 = seven days after the final chemotherapy cycle. No inferential statistical tests were performed.

Pain intensity, sensory disturbances, and daily-life impairment assessed with the Numerical Rating Scale (NRS) remained low throughout the study, with median and mean values between 1 and 2. No participant reported severe pain or disabling discomfort.

Local cooling of hands and feet during chemotherapy infusion was reported by four participants in the control group (66%) and two in the intervention group (40%). Cooling was applied using commercially available cooling pads or ice packs for approximately 15–20 minutes per session. No relevant differences in HFS incidence or severity were observed between the two groups with an overall low symptom intensity throughout treatment.

Patient diaries indicated good adherence to the intervention and consistent symptom documentation. Minor transient skin reactions such as mild erythema or warmth following cream application were reported by two participants and resolved spontaneously without discontinuation.

At the final visit, participants in the intervention group completed a short feasibility and satisfaction questionnaire. All rated the application procedure as “easy” or “very easy,” and 83% stated that they would continue using the product after study completion.

## Discussion

This pilot randomized controlled trial evaluated the feasibility and acceptability of Ice Plant Intensive Cream (Mesembryanthemum crystallinum) as a topical preventive intervention for chemotherapy-induced Hand–Foot Syndrome (HFS) in breast cancer patients treated with Doxorubicin and/or Docetaxel. Overall, among enrolled participants, the study procedures, assessment tools, and intervention were well tolerated and feasible under routine conditions. Once patients were enrolled, adherence and protocol compliance were high, with 100% of participants who started the intervention completing all planned visits and assessments, fulfilling the predefined feasibility threshold (≥ 80%).

Recruitment proved to be the most challenging aspect of this feasibility study. Of the 330 patients screened by the tumor board, only 67 (20.3%) received the relevant chemotherapy. This significant discrepancy between screening and eligibility numbers is not uncommon, as Unger et al. (17) demonstrated in their meta-analysis on the magnitude of structural, clinical, physician and patient barriers to cancer clinical trial participation. For future studies, it seems advisable to link screening to a therapy-related trigger (e.g., the preparation of the treatment plan) and to establish closer contact and earlier communication with the treating oncologists. Such alignment would help ensure that potentially eligible patients are approached before chemotherapy is initiated and would reduce loss of candidates due to narrow time windows for randomization and study inclusion. After further exclusions, 58 eligible patients remained. Only 13 out of 58 eligible patients (22%) consented to participate, despite the simplicity and low risk of the intervention. The main barriers included patient overload during initial diagnosis and the short interval between tumor board recommendation and chemotherapy initiation, which limited the available recruitment window. Similar recruitment constraints have been reported in other pilot studies in integrative oncology (18, 19). In addition, several patients stated during the phone call that they were under considerable emotional and organizational stress at the time of their initial cancer diagnosis. This underscores the problem of contact timing, which usually took place shortly after the diagnosis consultation. At the same time, this challenge highlights a major strength of integrative oncology within routine clinical care. Supportive consultations and integrative approaches often create space for concerns and fears that may not yet be visible during the chemotherapy information session or cannot be fully addressed in the limited time available. Through preventive strategies and individualized counseling, anxiety related to upcoming treatments can be reduced. Importantly, such processes unfold naturally in clinical everyday practice, whereas in a study setting this level of supportive interaction is inherently limited and more difficult to implement. While earlier contact alone may not necessarily improve recruitment, evidence suggests that patients are more likely to participate in clinical studies when their primary treating physician explicitly recommends taking part. One possible approach would therefore be to provide information earlier in the treatment trajectory — for example through written materials such as flyers or through a brief mention by the treating oncologist. A physician’s endorsement has been shown to strengthen trust, increase perceived relevance of the study, and reduce uncertainty during the decision-making process, all of which may enhance willingness to participate (20). In addition, several procedural adaptations may help improve recruitment in future feasibility-oriented studies. One potential strategy would be closer integration of the study team within the tumor board process, allowing treating physicians to introduce the study directly to potentially eligible patients as part of the initial therapeutic recommendation. This approach may reduce the perception of study participation as an additional burden. Another option would be to provide study information during the standard pre-chemotherapy informed consent process. Embedding study information into routine clinical communication may reduce the need for additional phone calls or extra visits, which are often experienced as stressful. Moreover, as preventive complementary interventions are frequently perceived as less relevant by patients who have not yet experienced treatment-related side effects, presenting such measures as an integrated component of the overall treatment plan may enhance their perceived relevance and acceptance.

We also discuss that closer interdisciplinary collaboration within the treating team, including stronger coordination with oncologists involved in treatment planning, may help streamline patient information processes and reduce perceived overload, thereby improving feasibility and acceptance in future studies.

Adherence to the Ice Plant Intensive Cream regimen was high, with all participants reporting daily use during chemotherapy. This suggests that topical supportive interventions can be realistically implemented in routine care without creating an additional burden for patients. Importantly, the consistently high adherence and acceptability observed in this study represent a key feasibility finding. Once the initial barrier to study entry was overcome, the intervention proved to be very well tolerated and easy to integrate into daily routines. These findings support the procedural feasibility of Ice Plant Intensive Cream as a topical supportive intervention. However, due to the design as a feasibility study, no conclusions can be drawn regarding its clinical efficacy or its suitability for inclusion in multimodal supportive-care approaches. An adequately powered confirmatory trial is needed to evaluate these questions.

Although the number of participants was too small to explore clinical trends, descriptive data suggest that HFS occurrence remained within the expected range for anthracycline- and taxane-based chemotherapy, with no grade 3 or 4 cases based on the mean grade per participant. The DLQI scores and diary entries were mostly in the low range, resulting in a pronounced floor effect. The mean values of the DLQI ranged between 0.6 and 1.2 (range 0-30) from T1 to T3. When assessing the HFS (range 0-4), the mean value was between 0.2 and a maximum of 0.5, and the values on the NRS scale for pain, sensory disturbances, and everyday impairments were between 1.2 and 1.9 (range 0-10) as shown in **Tables 6**, **7**. A qualitative evaluation was therefore not feasible. The individual stress on patients’ hands and feet was not taken into account. There can be significant differences depending on occupational activities and leisure activities. In addition, it cannot be ruled out that selection bias occurred, for example due to increased participation of patients with lower skin sensitivity. Furthermore, as already mentioned, diary compliance was very good, but it remains a subjective assessment and is prone to possible distortions. Due to the exploratory nature of this pilot study, the finding of low HFS intensity cannot be considered conclusive, but it is in line with previously reported trends for preventive skin care. This is also evident in a study on the use of urea cream for capecitabine-associated HFS. Wongkraisri et al. (21) demonstrated that although the occurrence cannot be prevented, the severity of the symptoms can be reduced.

These findings are consistent with a broader body of evidence supporting topical preventive strategies in supportive oncodermatology. Ren et al. demonstrated in a randomized controlled trial with 871 patients that prophylactic application of 10% urea cream significantly reduced the incidence of hand-foot skin reaction from 73.6% to 56.0% and delayed its onset in patients receiving sorafenib (12). Hofheinz et al. showed in a phase III trial with 152 patients that urea cream was superior to the antioxidant formulation Mapisal in preventing capecitabine-associated HFS, with incidence rates of 22.4% versus 39.5% (11). A meta-analysis of 16 randomized controlled trials with a total of 2,814 patients by Pandy et al. confirmed that urea cream and celecoxib significantly reduce HFS incidence across multiple systemic anticancer treatments (10). Recent randomized trials support topical anti-inflammatory strategies for preventing HFS. In the double-blind D-TORCH trial, 1% topical diclofenac significantly reduced both the incidence and severity of capecitabine-associated HFS compared with placebo, leading to fewer treatment interruptions (22).

Beyond HFS, the broader field of supportive oncodermatology has produced compelling evidence for topical preventive strategies across multiple anticancer treatment-related skin toxicities. In the STEPP trial, a preemptive topical regimen including moisturizers, sunscreen, topical corticosteroids, and doxycycline reduced the incidence of ≥grade 2 skin toxicities by more than 50% in patients receiving the EGFR inhibitor panitumumab compared with reactive treatment (23). Similarly, the J-STEPP trial confirmed that preemptive topical intervention significantly reduced ≥grade 2 skin toxicity from 62.5% to 21.3% (P<0.001) in patients treated with panitumumab (24). These findings, alongside the HFS-specific evidence cited above, collectively underscore the clinical relevance of proactive topical strategies across the spectrum of anticancer treatment-related dermatological toxicities, and highlight the importance of conducting adequately powered trials to establish the efficacy of novel agents such as Ice Plant Intensive Cream.

Given the small sample size of this pilot study, imbalances in baseline characteristics such as age and chemotherapy regimen between groups cannot be excluded and may have influenced the exploratory findings. Future confirmatory studies should consider stratified randomization to minimize such imbalances.

However, pilot studies are not designed to test efficacy hypotheses but rather to assess procedural feasibility, recruitment, and data completeness to inform future confirmatory research (25). From an implementation perspective, this study highlights both logistical and organizational challenges associated with conducting supportive-care trials within routine oncology workflows. Integrative interventions often require cross-disciplinary collaboration and clear communication between oncologists, nursing staff, and study coordinators to facilitate patient inclusion and consistent adherence (26). Future pilot designs should therefore emphasize early coordination with tumor boards, pre-screening procedures, and flexible timing to approach patients before chemotherapy initiation.

Moreover, fostering clinician awareness and patient communication about integrative supportive care could enhance recruitment and adherence in future trials. Recent analyses emphasize that structured education in integrative oncology is an emerging professional competency for oncology teams, facilitating safer and more consistent implementation of such interventions in daily care (18). This aspect is particularly relevant to ensure that supportive interventions such as Ice Plant Intensive Cream are presented credibly, integrated responsibly, and offered within evidence-based frameworks.

In summary, this pilot trial demonstrated that the study procedures, data collection instruments, and topical Ice Plant intervention are feasible and acceptable among enrolled participants. However, recruitment feasibility was not achieved, and these barriers highlight the need for optimized inclusion strategies and early patient engagement.

## Conclusion

The aim of this study was to assess the feasibility of conducting a larger confirmatory trial. The evaluation focused on recruitment, randomization, retention, adherence, and the acceptability of the prophylactic use of Ice Plant Intensive Cream for the prevention of Hand–Foot Syndrome (HFS). HFS is a frequent side effect of several chemotherapeutic agents that can substantially impair patients’ quality of life, while evidence for effective preventive or therapeutic options remains limited.

In this pilot study, recruitment feasibility was not achieved, whereas feasibility of the study procedures among enrolled participants, including retention, adherence, and data collection, was demonstrated. Once initiated, all participating patients completed the study, and the intervention was found to be safe and well tolerated. Recruitment, however, remained challenging, resulting in the inclusion of only 36% of the predefined target sample. These difficulties can largely be attributed to structural and timing-related factors. The findings provide valuable methodological insights for optimizing recruitment logistics and refining the design of future multicenter confirmatory trials evaluating topical preventive strategies for chemotherapy-induced skin toxicities.

## Data Availability

The original contributions presented in the study are included in the article/supplementary material. Further inquiries can be directed to the corresponding author.
