# Supplementary material for: Feasibility and acceptability of ice plant intensive cream in preventing hand-foot syndrome in breast cancer patients undergoing Doxorubicin and/or Docetaxel therapy: a randomized controlled pilot study
Source: Front Oncol. 2026 Aug 31;16:1758165. doi: 10.3389/fonc.2026.1758165 (PMC13572149; doi:10.3389/fonc.2026.1758165)
Supplement: Supplementary file 1 [file DataSheet1.pdf]

Supplementary Material 1

Dr. Hauschka MED Ice Plant Intensive Cream

Dr. Hauschka MED Ice Plant Intensive Cream

Product information: Dr. Hauschka MED products are cosmetic products (Article 2 of the EC Cosmetics Regulation). Cosmetic products are designed to care for and protect the skin and keep it in good condition.

Dr. Hauschka MED products are not applied directly to open skin and are not a substitute for medicinal ointments.

Dr. Hauschka MED Intensive Cream Mittagsblume is an intensive care product. It is suitable for extremely dry areas of skin, such as the crook of the arm or the back of the knee. Its rich formula provides intensive care, quickly restoring the skin's moisture content and stabilizing the skin barrier.

M-Questionnaire

General information

1. Identification number

Identification number:

Date:

Effects on the skin

2. How does your skin feel after application?

Very good	good	okay	moderate*	Very bad*	For *, please give reasons
◻	◻	◻	◻	◻	

Reasons:

---

3. The product offers a pleasant cooling effect.

Strongly agree	agree	Neither agree nor disagree	disagree*	Strongly disagree*	For *, please give reasons
◻	◻	◻	◻	◻	

Reasons:

Acceptance

4. Dr. Hauschka MED Ice Plant Intensive Cream reduces feelings of tightness in the skin.

◻ I do not feel any tension

(If this is the case, please do not answer this question and continue with question 6)

Strongly agree	agree	Neither agree nor disagree	disagree*	Strongly disagree*	For *, please give reasons
◻	◻	◻	◻	◻	

Reasons:

---

5. Dr. Hauschka MED Ice Plant Intensive Cream helps relieve dry skin.

◻ I do not have dry skin

(If this is the case, please do not answer this question and continue with question 7)

Strongly agree	agree	Neither agree nor disagree	disagree*	Strongly disagree*	For *, please give reasons
☐	☐	☐	☐	☐	

Reasons:

---

6. Dr. Hauschka MED Ice Plant Intensive Cream reduces itching of the skin.

☐ I do not have any itching

(If this is the case, please do not answer this question and continue with question 10)

Strongly agree	agree	Neither agree nor disagree	disagree*	Strongly disagree*	For *, please give reasons
☐	☐	☐	☐	☐	

Reasons:

---

7. The itching of the skin was quickly reduced.

Strongly agree	agree	Neither agree nor disagree	disagree*	Strongly disagree*	For *, please give reasons
☐	☐	☐	☐	☐	

Reasons:

8. The itching of the skin was significantly and sustainably reduced.

Strongly agree	agree	Neither agree nor disagree	disagree*	Strongly disagree*	For *, please give reasons
☐	☐	☐	☐	☐	

Reasons:

---

9. Dr. Hauschka MED Ice Plant Intensive Cream offers intensive skin care.

Strongly agree	agree	Neither agree/ nor disagree	disagree*	Strongly disagree*	For *, please give reasons
◻	◻	◻	◻	◻	

Reasons:

Tolerability

10. How well did your skin tolerate Dr. Hauschka MED Ice Plant Intensive Cream?

Very good	good	okay	moderate*	Very bad*	For *, please give reasons
◻	◻	◻	◻	◻	

Reasons:

Overall assessment

11. What is your overall evaluation of Dr. Hauschka MED Ice Plant Intensive Cream?

Very good	good	okay	moderate*	Very bad*	
◻	◻	◻	◻	◻	For *, please give reasons

Reasons:

12. Would you use Dr. Hauschka MED Ice Plant Intensive Cream again?

yes	no
◻	◻
